# Genome engineering in *Bacillus anthracis* using tyrosine site-specific recombinases

**DOI:** 10.1371/journal.pone.0183346

**Published:** 2017-08-22

**Authors:** Andrei P. Pomerantsev, Rita M. McCall, Margaret Chahoud, Nathan K. Hepler, Rasem Fattah, Stephen H. Leppla

**Affiliations:** Microbial Pathogenesis Section, Laboratory of Parasitic Diseases, National Institute of Allergy and Infectious Diseases, National Institutes of Health, Bethesda, Maryland, United States of America; University of Parma, ITALY

## Abstract

Tyrosine site-specific recombinases (T-SSR) are polynucleotidyltransferases that catalyze cutting and joining reactions between short specific DNA sequences. We developed three systems for performing genetic modifications in *Bacillus anthracis* that use T-SSR and their cognate target sequences, namely *Escherichia coli* bacteriophage P1 Cre-*loxP*, *Saccharomyces cerevisiae* Flp-*FRT*, and a newly discovered IntXO-*PSL* system from *B*. *anthracis* plasmid pXO1. All three tyrosine recombinase systems were used for creation of a *B*. *anthracis* sporulation-deficient, plasmid-free strain deleted for ten proteases which had been identified by proteomic analysis as being present in the *B*. *anthracis* secretome. This strain was used successfully for production of various recombinant proteins, including several that are candidates for inclusion in improved anthrax vaccines. These genetic tools developed for DNA manipulation in *B*. *anthracis* were also used for construction of strains having chromosomal insertions of 1, 2, or 3 adjacent *atxA* genes. AtxA is a *B*. *anthracis* global transcriptional regulator required for the response of *B*. *anthracis* virulence factor genes to bicarbonate. We found a positive correlation between the *atxA* copy number and the expression level of the *pagA* gene encoding *B*. *anthracis* protective antigen, when strains were grown in a carbon dioxide atmosphere. These results demonstrate that the three T-SSR systems described here provide effective tools for *B*. *anthracis* genome editing. These T-SSR systems may also be applicable to other prokaryotes and to eukaryotes.

## Introduction

Site-specific recombinases constitute a distinctive class of enzymes that possess the unique ability to both cleave and reseal DNA. Some of these recombinases function without cofactors and lead to precise, predictable and efficient genome modifications [1]. In particular, tyrosine site-specific recombinases (T-SSRs) have found widespread use in biotechnology and biomedical research. T-SSRs are DNA modifying enzymes that bind, cleave, exchange strands, and rejoin DNA at their respective, typically palindromic, target recognition sites [2]. Most T-SSRs are found in prokaryotes and bacteriophages where they perform a plethora of functions, including DNA integration and excision, plasmid copy number control, regulation of gene expression, chromosome segregation at cell division, and separation of multimeric circular DNAs [3]. In addition, T-SSRs are frequently encoded on yeast plasmids, where they complement the partitioning system to maintain high-copy numbers of the plasmids [4]. Among these, the bacteriophage P1 enzyme Cre and the yeast 2 μ- derived Flp recombinase are the enzymes most widely used in genome engineering. Using combinations of different T-SSRs in the same cell or organism has made it possible to establish increasingly sophisticated systems, and several different enzymes have been recently utilized in synthetic biology to build complex genetic circuits [2].

Accordingly, in the work continued here, we have created three T-SSR systems for performing genetic modifications in *Bacillus anthracis*: the *Escherichia coli* Cre-*loxP* and *Saccharomyces cerevisiae* Flp-*FRT* systems [5–7] and a newly developed *B*. *anthracis* IntXO-*PSL* system [8]. We previously used the Cre-*loxP* system to create the BH460 strain [9] having the following phenotypic characteristics: Spo0A-, pXO1-, pXO2-, NprB-, TasA-, Cam-, InhA1-, InhA2-, MmpZ-. This six protease-deficient, non-sporulating and avirulent strain was recommended as an effective host for recombinant protein production, typically yielding greater than 10 mg pure protein per liter of culture [9].

In this study, we describe work to further improve the BH460 strain by identification and deletion of additional secreted proteases. Functional and proteomic analysis of BH460 and its derivatives revealed four more proteases in the secretomes: CysP1, VpR, NprC and S41. The first three proteases were inactivated with the Flp-*FRT* system, producing the nine proteases mutant BH490. Deletion of the last S41 protease was performed with the IntXO-*PSL* system, yielding the ten proteases mutant BH500.

The three T-SSRs were also successfully used for sequential insertion of three copies of the *atxA* gene into the BH490 genome, as demonstrated here. The *atxA* gene encodes the pleiotropic regulator AtxA (anthracis toxin activator) [10] that regulates the expression of *B*. *anthracis* toxin genes in the presence of CO_2_. The two-step quantitative real-time PCR (qPCR) analysis demonstrated an increase in *atxA* and protective antigen (PA) gene *pagA* [11] transcription in the sequential recombinant strains, with maximum expression for the strain BH490A3 containing three *atxA* copies. Maximum PA production was also shown in the strain with three *atxA* genes. We plan to use the constructed multi-protease deficient strain BH490A3 for recombinant proteins overproduction with a promoter that responds to CO_2_ and AtxA activation.

## Materials and methods

### Materials

The *B*. *anthracis* strains, plasmids and genes inactivated or analyzed in this study are listed in Tables 1–3. All *B*. *anthracis* strains used here lack pXO2, are avirulent, are exempt from CDC Select Agent regulation, and are approved by NIAID to be used at BSL-2. Primers for PCR, primers and probes for qPCR, and T-SSR target sites that replaced the *B*. *anthracis* genes deleted in this study are listed in S1–S3 Tables.

**Table 1:** Bacterial strains used in this study.

*B*. *anthracis s*trains	Relevant characteristic(s)	Source or reference
Ames 35	*B*. *anthracis* (pXO1+, pXO2-), from Ames strain by removal of pXO2.	[7]
Ames 35 ΔAtxA	Ames 35 with *atxA* gene deleted	[12]
BH460	pXO1-, pXO2-, Spo0A-, NprB-, TasA-, Cam-, InhA1-, InhA2-, MmpZ-; total of five *loxP* sites in chromosome, six proteases inactivated	[9]
BH470	BH460 with *cysP1* knockout containing one *FRT* site	This work
BH480	BH470 with *vpR* knockout containing second *FRT* site in chromosome	This work
BH490	BH480 with *nprC* knockout containing third *FRT* site in chromosome	This work
BH490A	BH490 with the first *atxA* located upstream of the third *FRT* site	This work
BH490A2	BH490A with the second *atxA* located upstream of the first *atxA* copy; sixth *loxP* site is located between two *atxA*	This work
BH490A3	BH490A2 with the third *atxA* located downstream of the sixth *loxP* site and between two originally inserted *atxA*; contains one *PSL* site in chromosome	This work
BH500	BH490 with *s41* knockout containing one *PSL* site in chromosome	This work

**Table 2:** Plasmids used in this study.

Plasmid	Relevant characteristic(s)	Source or reference
pSC	Contains multiple restriction site flanked by two direct *loxP*; pSC has permissive and restrictive temperatures of 30°C and 37°C for *B*. *anthracis*, respectively. Amp^r^ in *E*. *coli*, Em^r^ both in *E*. *coli* and *B*. *anthracis*	[7]
pSC-A	*Xho*I/*Zra*I PCR fragment containing *atxA* gene was inserted into pSC *Xho*I/*Sma*I sites	This work
pSCF	Contains multiple restriction site flanked by two direct *FRT*; pSCF has permissive and restrictive temperatures of 30°C and 37°C for *B*. *anthracis*, respectively, and can be easily isolated from *E*. *coli* strains grown at 37°C; Amp^r^ in *E*. *coli*, Em^r^ both in *E*. *coli* and *B*. *anthracis*.	[6]
pSCF-2183L	*Bst*Z17I/*Xho*I PCR fragment containing upstream fragment of *nprC* gene was inserted into pSCF	This work
pSCF-2183LA	*Xho*I/*Zra*I PCR fragment containing *atxA* gene was inserted into pSCF-2183L *Xho*I/*Sma*I sites	This work
pSCF-2183R	*Xma*I/*Sac*I PCR fragment containing downstream fragment of *nprC* gene was inserted into pSCF	This work
pSCP	Contains multiple restriction site flanked by two direct *PSL*; pSCP has permissive and restrictive temperatures of 30°C and 37°C for *B*. *anthracis*, respectively, and can be easily isolated from *E*. *coli* strains grown at 37°C; Amp^r^ in *E*. *coli*, Em^r^ both in *E*. *coli* and *B*. *anthracis*.	[8]
pSCP-A	*Xho*I/*Zra*I PCR fragment containing *atxA* gene was inserted into pSCF *Xho*I/*Zra*I sites	This work
pCrePAS2	Contains entire Flp recombinase gene under the control of *pagA* promoter; pCrePAS2 has permissive and restrictive temperatures of 30°C and 37°C for *B*. *anthracis*, respectively, and can be easily isolated from *E*. *coli* strains grown at 37°C.	[6]
pFPAS	Contains entire Flp recombinase gene under the control of *pagA* promoter; pFPAS has permissive and restrictive temperatures of 30°C and 37°C for *B*. *anthracis*, respectively, and can be easily isolated from *E*. *coli* strains grown at 37°C; Sp^r^ both in *E*. *coli* and *B*. *anthracis*.	[6]
pIntPAS	Contains entire IntXO recombinase gene under the control of pagA promoter; pIntPAS has permissive and restrictive temperatures of 30°C and 37°C for *B*. *anthracis*, respectively, and can be easily isolated from *E*. *coli* strains grown at 37°C; Sp^r^ both in *E*. *coli* and *B*. *anthracis*.	[8]
pGEM-T Easy	Cloning vector for PCR products; Ap^r^ in *E*. *coli*.	Promega
pUC4-ΩKM2	pUC4 carrying an Ω-*kan* element with kanamycin resistance marker; Km^r^ in *E*. *coli* and *B*. *anthracis*.	[6]
pYS5	*B*. *anthracis* expression vector. Transcription of *pag* is under control of truncated *pag* promoter.	[13]
pPAGP	pYS5 with *SnaBI/*Hind*III* small fragment replaced by *SnaBI/*Hind*III* PCR fragment amplified with pagPF/pagPR primer pair from *B*. *anthracis* Ames 35 pXO1plasmid	This work
pPAGK	pYS5 derivative (*neo* gene replaced with Ω-*kan* from pUC4-ΩKM2, transcription of *pag* is under control of full length *pag* promoter).	This work
pSJ115	Contains *B*. *anthracis lef* gene instead of the *pag* gene in pYS5. Retains the *pag* signal sequence gene.	[14]
pSJ136EFOS	Contains *B*. *anthracis cya* gene instead of the *lef* gene in pSJ115. Retains the *pag* signal sequence gene.	[9]
pUTE29-*htrA*	The *B*. *anthracis htrA* gene amplified with HAF/HAR primers pair was inserted into pGEM-T Easy. The *Sal*I-*Bam*HI fragment of pGEM-T Easy-*htrA* containing the *htrA* gene was inserted between *Sal*I, *Bam*HI sites of pUTE29 [15] producing pUTE29-*htrA*.	This work

**Table 3:** *B*. *anthracis* Ames ancestor strain genes inactivated or analyzed in this study.

Protein	Gene	Function/Name	Locus Tag
CysP1	*cysP1*	Putative cysteine protease	GBAA_1995
NprC	*nprC*	Neutral metalloprotease	GBAA_2183
VpR	*vpR*	Minor extracellular protease	GBAA_4584
S41	*s41*	Serine protease	GBAA_5414
HtrA	*htrA*	Serine protease	GBAA_3660
AtxA	*atxA*	Transcriptional regulator	GBAA_RS29060
EF	*cya*	Edema factor	GBAA_RS29035
LF	*lef*	Lethal factor	GBAA_RS29135
PA	*pag*	Protective antigen	GBAA_RS29110
RpoB	*rpoB*	RNA polymerase subunit β	GBBA_0102
DnaJ	*dnaJ*	Chaperone	GBBA_4538
GyrB	*gyrB*	DNA gyrase subunit B	GBBA_0005

### Bacterial growth conditions and phenotypic characterization

*Escherichia coli* strains were grown in Luria-Bertani (LB) broth and used as hosts for cloning. LB agar was used for selection of transformants [16]. *B*. *anthracis* strains were also grown in LB, FA [13] or NBY [12] liquid medium (ambient air). For growth with CO_2_, the NBY medium was supplemented with 0.8% (w/v) NaHCO_3_ and the air was supplemented with 15% CO_2_. Antibiotics (Sigma-Aldrich, St. Louis, MO) were added to the medium when appropriate to give the following final concentrations: ampicillin (Ap), 100 μg/ml (only for *E*. *coli*); erythromycin (Em), 400 μg/ml for *E*. *coli* and 10 μg/ml for *B*. *anthracis*; spectinomycin (Sp), 150 μg/ml for both *E*. *coli* and *B*. *anthracis*; kanamycin (Km), 15 μg/ml and tetracycline (Tc), 5 μg/ml for *B*. *anthracis*. SOC medium (Quality Biologicals, Inc., Gaithersburg, MD) was used for outgrowth of transformation mixtures prior to plating on selective media to isolate transformants.

### DNA isolation and manipulation

Preparation of plasmid DNA from *E*. *coli*, transformation of *E*. *coli*, and recombinant DNA techniques were carried out by standard procedures [16]. *E*. *coli* SCS110 competent cells were purchased from Agilent Technologies (Santa Clara, CA), and *E*. *coli* TOP10 competent cells were purchased from Life Technologies (Grand Island, NY). Recombinant plasmid construction was carried out in *E*. *coli* TOP10 cells. Plasmid DNA from *B*. *anthracis* was isolated according to the *Plasmid Protocol*: *Purification of Plasmid DNA from Bacillus subtilis* (QIAGEN Inc., Valencia, Calif.). Chromosomal DNA from *B*. *anthracis* was isolated with a Wizard genomic purification kit (Promega, Madison, WI) in accordance with the protocol for isolation of genomic DNA from Gram-positive bacteria. *B*. *anthracis* was electroporated with unmethylated plasmid DNA isolated from *E*. *coli* SCS110 (*dam-/dcm-*) (Stratagene, San Diego, CA). Electroporation-competent *B*. *anthracis* cells were prepared and transformed as previously described [7].

Restriction enzymes, T4 ligase, T4 DNA polymerase, and alkaline phosphatase were purchased from New England BioLabs (Ipswich, MA). The pGEM-T Easy vector system (Promega) was applied for PCR fragment cloning. Phusion high-fidelity DNA polymerase from New England BioLabs was used for fragment PCR. OneTaq^®^ 2X Master Mix with Standard Buffer (New England BioLabs) were used for routine DNA analysis. All constructs were verified by restriction enzyme digestion and/or DNA sequencing. Plasmid and chromosomal DNAs were sequenced using primers listed in S1 Table. Primers for sequencing and PCR were synthesized by Integrated DNA Technologies (IDT, Inc. Coralville, IA). Sequences were determined using a primer walking strategy (Macrogen, Rockville, MD). Sequence data were assembled using the Vector NTI software (Life Technologies, Carlsbad, CA). The National Center for Biotechnology Information (NCBI) BLAST and FASTA programs (http://www.ncbi.nlm.nih.gov) were used for homology searches in GenBank and nonredundant protein sequence databases. Predicted protein motifs were analyzed using SignalP software version 3.0 for prediction of signal sequences (http://www.cbs.dtu.dk/services/SignalP) and TMpred software for prediction of transmembrane regions and orientation (http://www.ch.embnet.org/software/TMPRED_form.html).

### Recombinase systems used for preparation of mutants

Genetic modifications in the *B*. *anthracis* genome were generated with the Cre-*loxP* system as previously described [7], employing plasmids we designated generically as pSC, for single-crossover plasmid, together with pCrePAS2 for Cre recombinase production [6]. Deletions and insertions were also generated with the Flp-*FRT* system, employing plasmids pSCF (an analog of the pSC plasmid) and pFPAS (a plasmid with all the characteristics of pCrePAS2, but containing the *flp* gene instead of the *cre* gene) as previously described [6].

**Fig 1:**
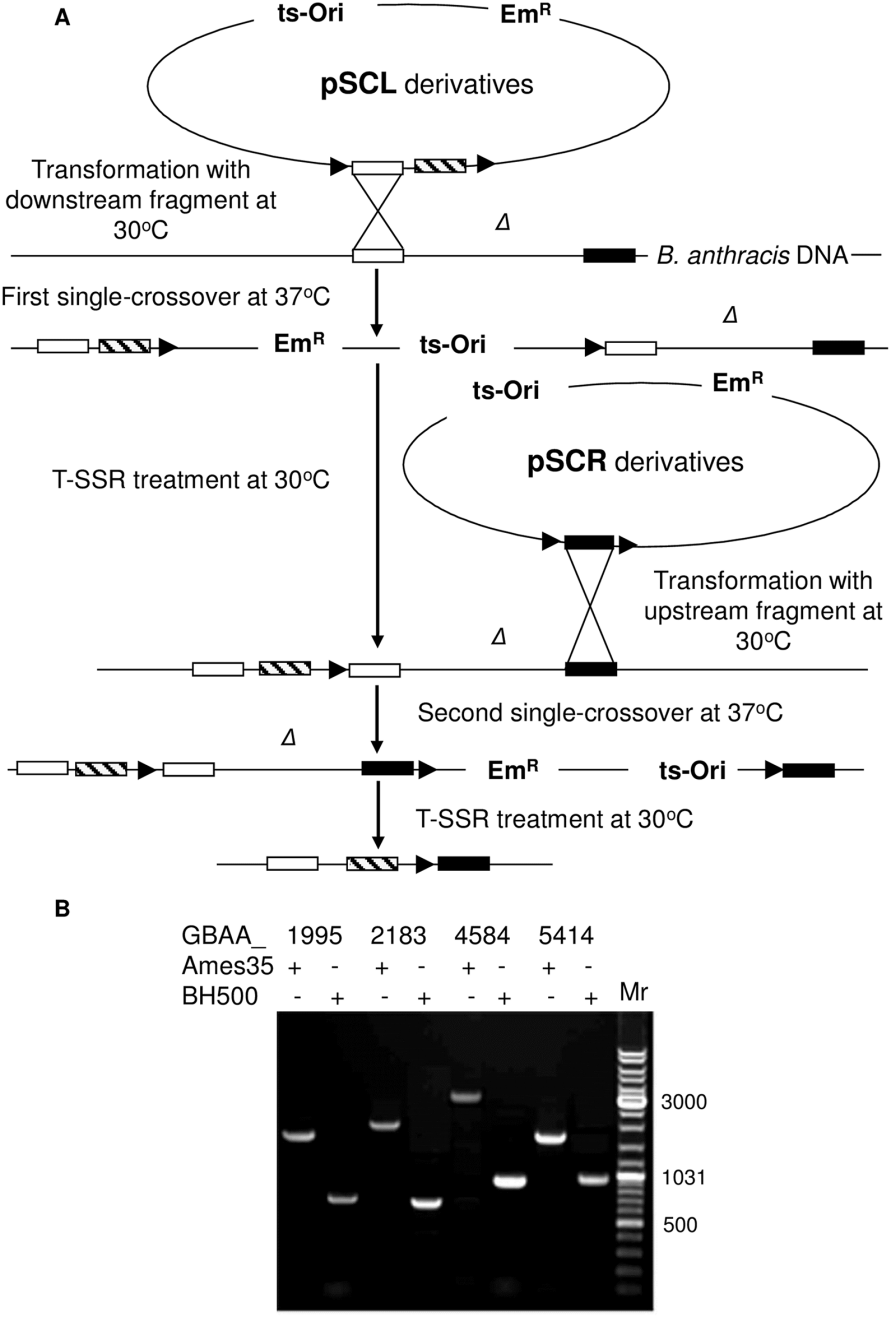
T-SSR system used for deletion/insertion of DNA fragments in *B*. *anthracis* genome. (A) Scheme of the T-SSR application. White and black rectangles are fragments flanking *Δ*, the DNA fragment to be deleted, while the striped rectangle is the DNA fragment to be inserted in place of *Δ*. Black triangles represent target sequences *loxP*, *FRT* or *PSL* for Cre, Flp or IntXO T-SSR, respectively. The detailed description of the application of Cre-*loxP*, Flp-*FRT* and IntXO-*PSL* were described previously [6–8] and in the Results section. (B) PCR verification of deletions in *cysP1*, *nprC*, *vpR* and *s41* protease genes with corresponding primer pairs 1995seqF/R, 2183 seqF/R, 4584 seqF/R and 5414 seqF/R. GenBank accession numbers for proteases and strains used to verify the retention of specific segments are indicated at the top of the gel. Mr, GeneRuler DNA ladder mix for size determination (e.g., 3,000 bp).

A new IntXO-*PSL* recombinase system based on the recently identified IntXO recombinase of *B*. *anthracis* [8] was created for use in a manner analogous to that of the Flp-*FRT* system described above. The plasmid pSCP is a shuttle plasmid with all the characteristics of pSCF, except that it contains repeats of the 37-bp *PSL* sequence instead of the *FRT* sequence. An analog of the pFPAS plasmid that we created and designated as pIntPAS is a shuttle vector with all the characteristics of pFPAS, except that it contains the IntXO recombinase gene instead of *flp*. All three T-SSRs described above were used for genetic deletions or insertions in accordance with the scheme presented in Fig 1, as will be detailed later. Sequences and maps of the plasmids described in this section are available upon request.

### Identification of additional proteases in the protease-deficient strains BH460 and BH480

Both BH460 and BH480 strains (Table 1) were grown overnight in FA medium and centrifuged at 10,000 g for 15 min. The supernatants were filter sterilized using a Pellicon cassette system (EMD Millipore, Billerica, MA) with Durapore 0.22-mm membranes and concentrated with Ultracel-10K membrane (Amicon Ultra-4 centrifugal filters, Merck Millipore Ltd. Tullagreen, IRL). The concentrated supernatants were loaded onto a hydroxyapatite column (Bio-Rad, Hercules, CA) that was pre-equilibrated with 0.02 M potassium phosphate and 0.1 M NaCl buffer (pH 7.0). Proteins were eluted at 1 ml/min in a 0.02–0.25 M potassium phosphate gradient and protein-containing fractions were analyzed for proteolytic activity. Casein protease activities of the supernatants were quantified by the Enz Chek Protease Assay Kit E-6638 (Invitrogen, Thermo Fisher Scientific, Grand Island, NY). For measurement of the protease activity, each 100 μL reaction mixture contained 5 μg/ml of BODIPY FL casein and 5 μl supernatant in 10 mM Tris-HCl (pH 7.8). Reaction mixtures were incubated in the dark at 37°C for 60 min. Fluorescence was measured with a Wallace 1420 VICTOR 96-well plate reader (Perkin Elmer, Boston, MA) with excitation at 485 nm and emission at 530 nm. Fractions with proteolytic activities were loaded on 4–20% SDS PAGE (Life Technologies). Every band was marked and sent for LC MS-MS analysis at the Research Technologies Branch, NIAID/NIH Twinbrook I Facility, Rockville, MD.

### Construction of vectors for protease gene inactivation and preparation of mutants

*B*. *anthracis* BH460 (derived from the pXO1-, pXO2- Ames 33 strain) was used for genetic manipulations. The GenBank database (GenBank Accession No. for the Ames strain is NC_003997) was analyzed for the identification of target genes and for the corresponding primer design. To inactivate CysP1, VpR and NprC proteases we amplified upstream and downstream fragments with corresponding primer pairs (S1 Table) and inserted them into pSCF to produce recombinant plasmids used for integration by homologous recombination with chromosomal DNA in accordance with the scheme (Fig 1). To inactivate the S41 gene, the same procedure was performed and amplified fragments were inserted into the pSCP plasmid for further integration. Subsequent transformation of the recombinant strains either with pFPAS or pIntPAS plasmid eliminated the protease genes and produced strains BH470, BH480, BH490 and finally BH500 (Table 1).

PCR fragments containing either *FRT* or *PSL* sites within mutated genes were amplified and sequenced using primers listed in S1 Table (1995seqF/1995seqR, 2183seqF/2183seqR, 4584seqF/4584seqR, and 5414seqF/5414seqR).

### Protein purification and verification

*B*. *anthracis* toxin components and HtrA protease were expressed in strain BH500 from plasmids pSJ136EFOS (EF), pSJ115 (LF), pYS5 (PA) and pUTE29-*htrA* (Table 2). The strains containing plasmids were grown in FA medium containing 20 μg/ml of kanamycin (or 10 μg/ml tetracycline for pUTE29-*htrA*) at 37°C for 14 h, largely following procedures previously used for production of LF [14]. The cultures were cooled and, except in the case of HtrA, supplemented with 2 μg/ml of AEBSF [4-(2-Aminoethyl)-benzenesulfonylfluoride HCl] (US Biological, Swampscott, MA), and then centrifuged at 4550 *g* for 30 min. All subsequent steps were performed at 4°C. The supernatants were filter sterilized and supplemented with 5 mM EDTA. Solid ammonium sulfate was added to the supernatants to obtain 40% saturation. Phenyl-Sepharose Fast Flow (low sub) (GE Healthcare Life Sciences, Uppsala, Sweden) was added and supernatants gently mixed in 4°C for 1.5 h. The resins were collected on porous plastic funnels (BelArt Plastics, Pequannock, NJ) and washed with buffer containing 1.5 M ammonium sulfate, 10 mM Tris HCl, and 1 mM EDTA (pH 8.0). The proteins were eluted with 0.3 M ammonium sulfate, 10 mM Tris HCl, and 1 mM EDTA (pH 8.0), precipitated by adding an additional 30 g ammonium sulfate per 100 mL eluate, and centrifuged at 18,370 *g* for 20 min. The proteins were dissolved and dialyzed against 5 mM HEPES, 0.5 mM EDTA (pH 7.5). The dialyzed samples were applied to a Q-Sepharose Fast Flow column (GE Healthcare Life Sciences) and eluted with a 0–0.5 M NaCl gradient in 20 mM Tris–HCl, 0.5 mM EDTA (pH 8.0). The protein-containing fractions identified by SDS-PhastGel analysis were purified on a column of ceramic hydroxyapatite (Bio-Rad Laboratories) with a gradient of 0.02–1.0 M potassium phosphate containing 0.1 M NaCl (pH 7.0). The fractions containing proteins were dialyzed overnight against 5 mM HEPES and 0.5 mM EDTA, pH 7.5, concentrated as necessary, frozen, and stored at −80°C. The molecular masses of purified toxin components were confirmed by liquid chromatography-electrospray ionization mass spectrometry (LC-ESI-MS) using an HP/Agilent 1100 MSD instrument (Hewlett Packard, Palo Alto, CA) at the National Cancer Institute (Frederick, MD) as described previously [9]. The N-terminal sequence of HtrA was determined using a gas-phase sequencer at the Research Technologies Branch, NIAID/NIH Twinbrook I Facility (Rockville, MD). All purified proteins were quantified by Ultrospec 2100 UV/Visible spectrophotometer (Amersham Pharmacia Biotech Inc., Piscataway, NJ) at 280 nm.

### SDS-PAGE protein analysis

**Fig 2:**
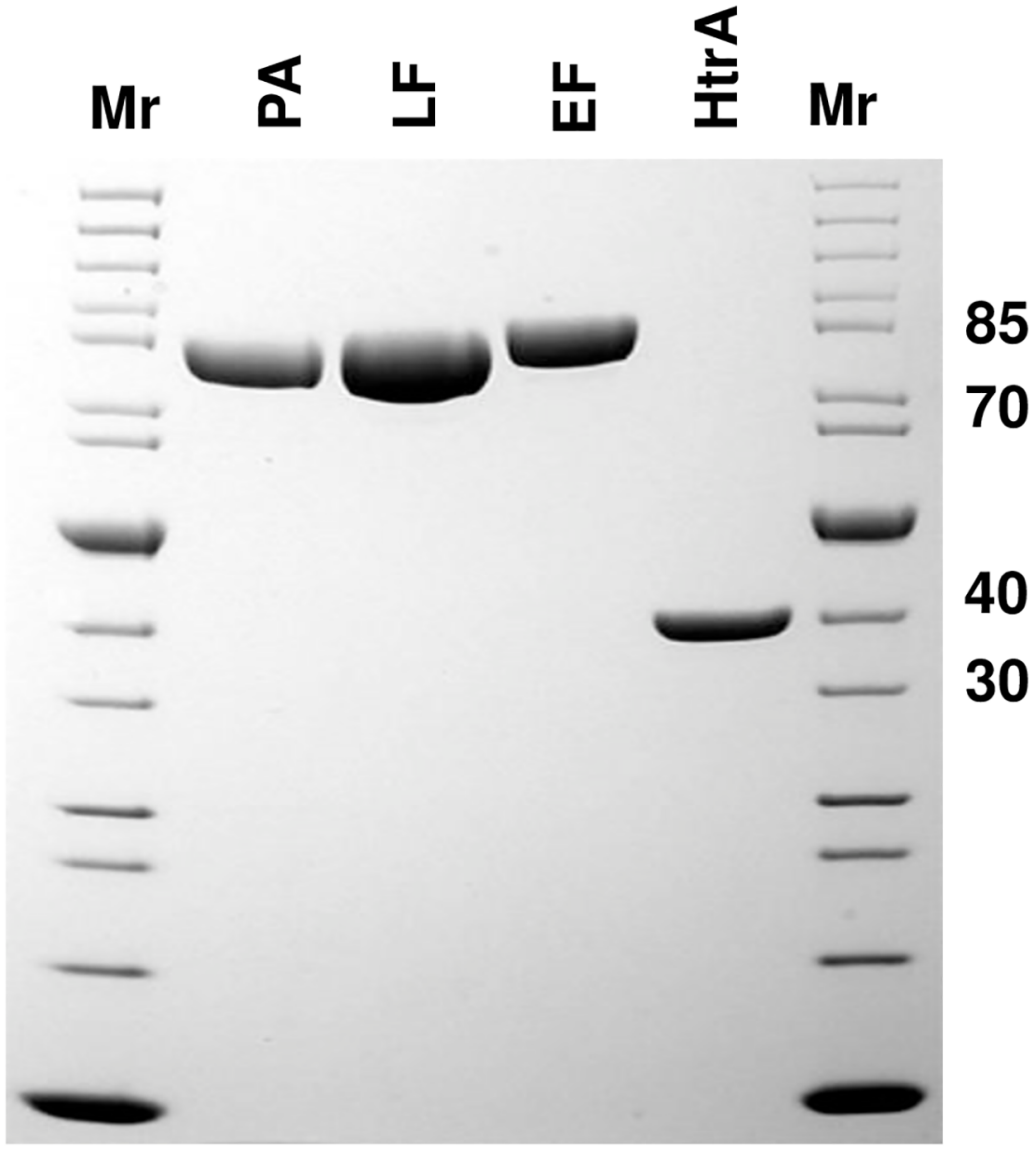
SDS-PAGE analysis of the PA, LF, EF and HtrA proteins (Coomassie stained). Proteins were purified from BH500 strains containing pYS5, pSJ115, pSJ136EFOS and pUTE29-*htrA* plasmids, respectively. The strains were grown in FA medium with 20 μg/ml of kanamycin (or 10 μg/ml tetracycline for pUTE29-*htrA*) at 37°C for 14 h. Protein purification was performed following procedures described in the Materials and Methods section. Mr—PageRuler unstained protein ladder mix for size determination (e.g., 85 kDa).

Proteins were analyzed by Bolt^®^ Bis-Tris Plus 4–12% gradient gels (Life Technologies) with the Bolt^®^ MES as running buffer (B0002, Life Technologies). Samples were prepared by mixing 10 μl of SDS loading buffer (161–0747, Bio-Rad) with 40 μl of protein samples and boiling for 5 min at 95°C. Samples of 5 μg of the protein were loaded on the gel (Fig 2). Electrophoresis was run using the Bolt Mini Gel tank (Life Technologies) at 165 volts for 35 min.

### Construction of vectors for *atxA* gene insertion and preparation of mutants

**Fig 3:**
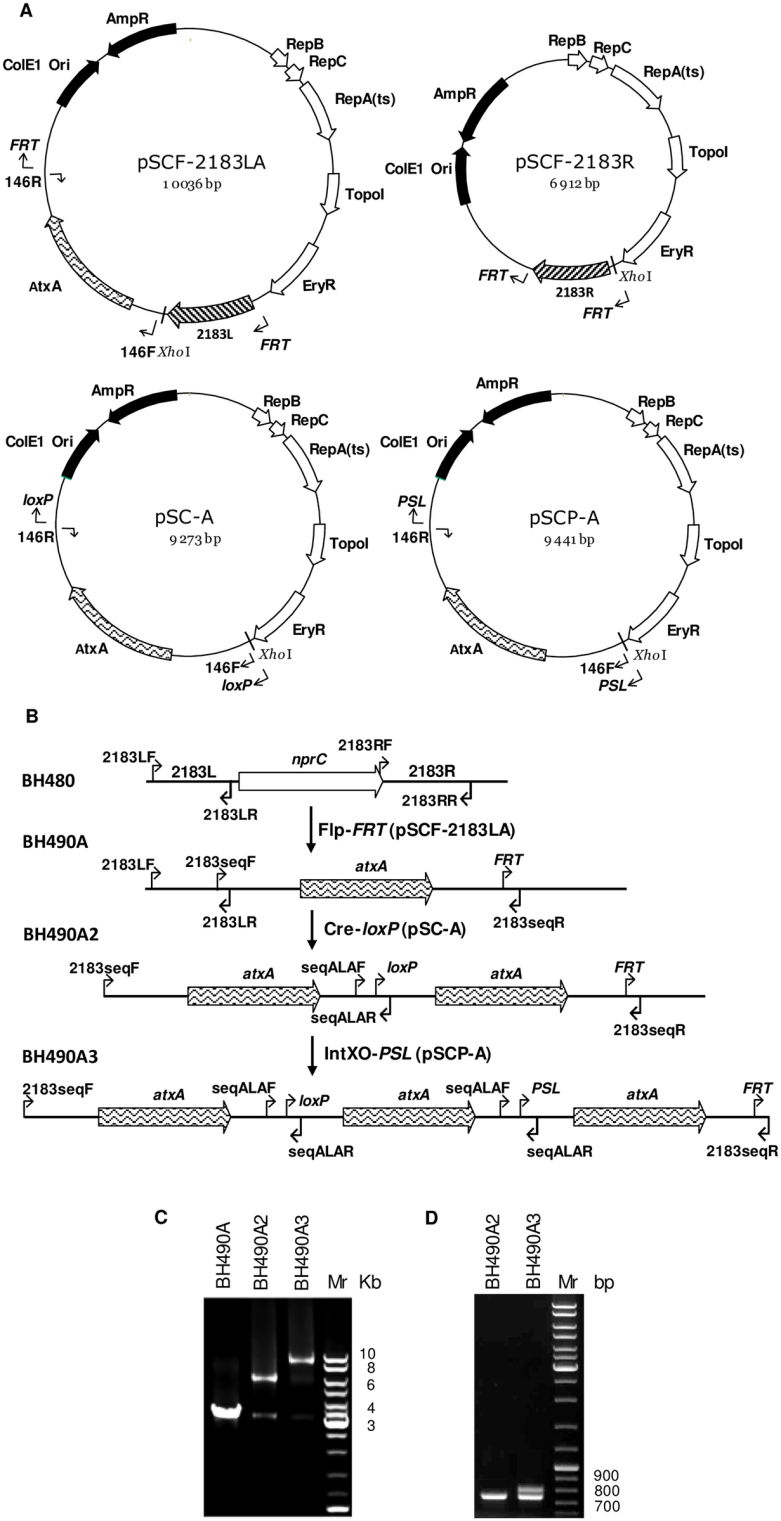
Creation of *B*. *anthracis* strains containing variable numbers of *atxA* genes replacing the *nprC* gene. (A) Plasmids used for sequential *atxA* gene insertions. (B) Scheme indicating sequential *atxA* gene insertions: *nprC* gene was replaced by the first *atxA* gene using Flp-*FRT* system with pSCF-2183LA plasmid as a donor of *atxA*; the second *atxA* was added from pSC-A plasmid with Cre-*loxP* system; and the third *atxA* copy was inserted with IntXO-*PSL* system from pSCP-A plasmid. (C) PCR confirmation of *atxA* gene insertions into genome of *B*. *anthracis* (primers 2183seqF/2183seqR). Strains with one, two, and three copies of *atxA* are indicated on the top of the gel. (D) PCR confirmation of *atxA* intergenic regions in strains with double and triple *atxA* genes (primers seqALAF/ALARseqR).

A PCR fragment containing the *atxA* promoter, ORF, and 3’ terminator was amplified with primer pair 146F/146R (S1 Table). The fragment was inserted along with the upstream fragment of the *nprC* gene amplified by primers 2183LL/2183LR (S1 Table) into the pSCF plasmid to produce the pSCF-LA construct. The same *atxA* fragment was inserted into the pSC and pSCP vectors to produce the pSC-A and pSCP-A plasmids, respectively (Fig 3A). The first insertion of the *atxA* gene was performed by the Flp-*FRT* T-SSR in accordance with the scheme of Fig 1. The plasmid pSCF-R was used to complete deletion of the *nprC* gene. Subsequent insertions of second and third copies of *atxA* were performed by homologous recombination within the *atxA* sequence using the Cre-*loxP* and IntXO-*PSL* T-SSRs. The scheme used in preparation of these BH490 strain variants containing one, two and three copies of the *atxA* gene and confirmation of structures by PCR analysis using the 2183seqF/2183seqR and seqALAF/seqALAR primer pairs is shown in Fig 3.

### Construction of pPAGK plasmid

**Fig 4:**
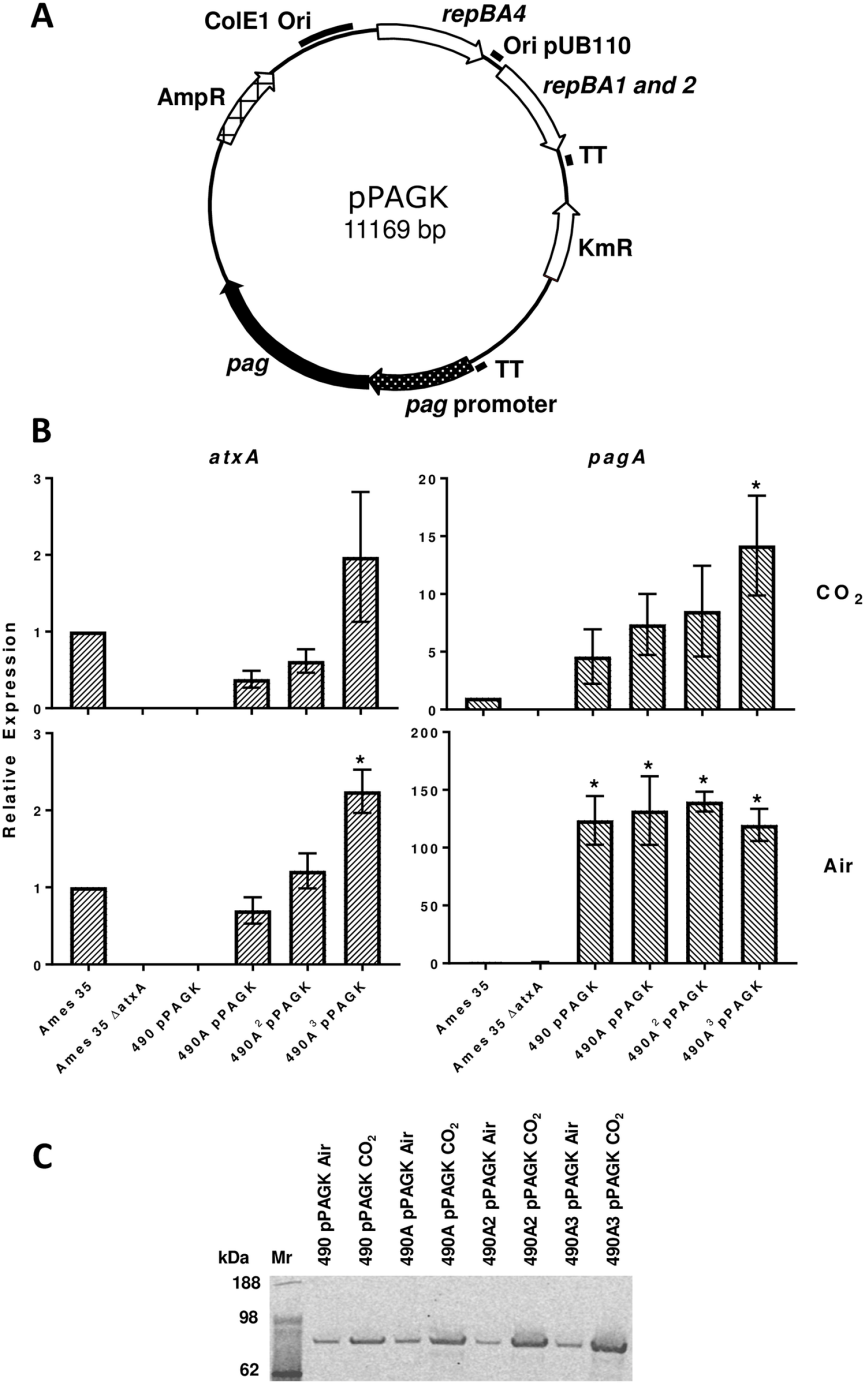
Increased *atxA* copy number enhances *pagA* gene transcription and PA content in the *B*. *anthracis* secretome. (A) Genetic structure of pPAGK plasmid. TT—T4 phage transcription terminator. (B) Active transcription of *atxA* and *pagA* in BH490 derivatives containing the pPAGK plasmid. Two-step qPCR results of *atxA* and *pagA* transcription respectively from cultures grown in NBY broth with 0.8% NaHCO_3_ in 15% CO_2_ (top panels) and in air (bottom panels). Relative expression represents the 2^-ΔΔCt^ (RQ) value normalized to Ames 35 and evaluated with three reference genes: *rpoB*, *gyrB*, and *dnaJ*. Values reported are the mean relative expression ± standard error of the mean calculated from ExpressionSuite and GraphPad Prism software. An asterisk indicates a significant difference (*p*<0.05) from an unpaired t-test compared to Ames 35. (C) Western blot analysis of PA production in strains grown in the same conditions as in panel B.

To create plasmids containing the full length promoter of the *pag* gene (1260 bp) as originally identified by Uchida et al. [10] (Table 2), we first amplified that region with the pagPF/pagPR primer pair from the *B*. *anthracis* Ames 35 pXO1 plasmid and inserted it into the large *SnaBI/*Hind*III* fragment of pYS5. The resulting pPAGP plasmid was then cut with *Bbs*I/*Sna*BI. The sticky *Bbs*I site end was filled (blunted) with T4 DNA polymerase and the linearized plasmid was ligated to the *Sma*I fragment of pUC4-ΩKM2 containing *Ω*-kan element with the kanamycin resistance marker flanked by two bacteriophage T4 transcriptional terminators. The *kan* gene of the resulting pPAGK plasmid was inserted in opposite orientation to the *pag* gene and its promoter (Fig 4). As a result, the short *pag* promoter (162 bp) of the pYS5 plasmid was replaced with the full length *pag* promoter (1260 bp) in pPAGK.

### Preparation of samples for RNA and protein analysis

BH490 strain variants (A, A2, A3) containing the pPAGK plasmid were inoculated into NBY broth containing kanamycin and grown overnight at 37°C in air or with 0.8% (w/v) sodium bicarbonate in 15% CO_2_. The overnight cultures were diluted to A_600_ of 0.05 and grown at 37°C in the same media until A_600_ = 2.0. The bacterial cultures were then centrifuged at 10,000 g for 10 min at 4°C. Supernatants and pellets were separated, frozen on dry ice and stored at -80°C for further analysis of PA and RNA content. Ames 35 and Ames 35 ΔAtxA strains were grown in parallel as controls.

### RNA isolation and purification

Bacterial cell pellets were suspended in RNA*later* stabilization solution (Ambion, Life Technologies) and incubated at room temperature for 1 h. After incubation, suspensions were centrifuged at 12,000 g for 5 min at 4°C and pellets were flash frozen on dry ice and stored at -80°C. Pellets were thawed on ice and washed with RNase-free water and re-pelleted by centrifugation at 15,000 g for 5 min at 4°C. The pellets were re-suspended in Buffer RLT from the RNeasy Mini Kit (Qiagen, Germantown, MD) and shaken with Lysing Matrix B (MP Biomedicals, Santa Ana, CA) for 20 sec at 6.0 M/s using FastPrep-24 (MP Biomedicals). The bacterial lysates were treated according to manufacturer’s instructions and incubated with TURBO DNase (Ambion, Life Technologies), again following manufacturer’s instructions, for 1 h at 37°C. The partially cleaned RNAs were purified again using the RNeasy Mini Kit, treated again with TURBO DNase, purified once more with the RNeasy Mini Kit, and then cleaned up using the RNA Cleanup procedure from the RNeasy Mini Kit protocol. We established that our protocol requiring two TURBO DNase incubations ensured elimination of contaminating genomic and plasmid DNA (S5 Table). RNA integrity numbers were determined using an Agilent 2100 Bioanalyzer (Agilent Technologies). Purity and concentration of the RNA was determined using a Nandrop ND-1000 spectrophotometer (Thermo Scientific, Wilmington, DE). The RNA samples were stored at -80°C before cDNA synthesis.

### qPCR analysis

Purified RNAs were converted to cDNA with Superscript^™^ Vilo^™^ cDNA Synthesis Kit (Invitrogen, Thermo Fisher Scientific) according to the manufacturer’s instructions with a minor modification: 12.5 μg of RNA in 100 μL of total reaction volume was incubated at room temperature for 10 min, 42°C for 2 h, and terminated at 85°C for 5 min. The synthesized cDNA was stored in -20°C until used for analysis.

The transcription levels of *pagA* and *atxA* genes were assayed by qPCR with three reference genes in accordance with the MIQE guidelines [17]: *rpoB*, *gyrB*, and *dnaJ* (Table 3). The cDNA was amplified using TaqMan Fast Advanced Master Mix (Applied Biosystems, Thermo Fisher Scientific) with IDT-prepared primers and Applied Biosystems-prepared probes (S2 Table). The final concentration of primers and probes were 300 nM and 250 nM, respectively. Reactions were plated in MicroAmp Fast Optical 96-Well Reaction Plates (Applied Biosystems) in 20 μL reactions with 20 ng of cDNA and in three technical replicates and assayed with Applied Biosystems 7500 Fast Real Time PCR System. Cycling conditions were as follows: 2 min at 50°C, 20 sec at 95°C and 40 cycles of 3 sec at 95°C and 30 sec at 60°C. Data were analyzed using the ΔΔC_t_ method and normalized to Ames 35 using Applied Biosystems ExpressionSuite Version 1.0.3 software (https://www.thermofisher.com/us/en/home/technical-resources/software-downloads/expressionsuite-software.html). GraphPad Prism 7.01 software (http://www.graphpad.com/scientific-software/prism/) was used for graphing and statistical analysis. Data were analyzed by unpaired t-tests comparing all strains to the Ames 35 control and are presented as the mean ± standard error of the mean. Statistical significance was considered as *p*<0.05. All qPCR graphs represent combined data from three biological replicates.

### Analysis of PA production under control of AtxA and carbon dioxide

Supernatant samples from bacterial culture samples grown either in air or CO_2_ were loaded onto Bolt 4–12% Bis-Tris Plus gels and run as described previously in the Materials and Methods section (paragraph “SDS-PAGE protein analysis”). Proteins were transferred to nitrocellulose paper using the iBlot 2 Dry Blotting System (Invitrogen, Thermo Fisher Scientific). After transferring, the blot was blocked for 45 min at room temperature in Odyssey Blocking Buffer (PBS) (LI-COR Biosciences) and incubated with anti-PA rabbit primary antibody 1:2000 dilution [18] in the blocking buffer + 0.05% Tween 20. After overnight incubation at room temperature, membrane was rinsed 3 times for 10 min in 1X PBST (PBS + 0.05% Tween 20) and washed another 3 times with 1X PBST for 5 min. An anti-rabbit HRP secondary antibody (Rockland, Limerick, PA) was diluted 1:10,000 in in the blocking buffer + 0.05% Tween 20 as before and membrane was incubated for 45 min at room temperature. Membrane was then rinsed as described before and imaged.

## Results

### Four additional proteases were identified in, and deleted from, strain BH460

Our previous work identified and then deleted several proteases secreted by *B*. *anthracis*, resulting in strain BH460, lacking 6 proteases (S3 Table). To extend this analysis, supernatants collected from BH460 cultures were separated by hydroxyapatite chromatography, and peaks having protease activity were analyzed by MS/MS. The two active fractions from BH460 were found to contain two caseinolytic proteases, CysP1 and VpR (S1 Fig). CysP1 belongs to a transglutaminase/protease-like superfamily having a conserved Cys/His/Asp catalytic triad. The minor extracellular protease VpR belongs to the S8 family of serine proteases characterized by the Asp/His/Ser catalytic triad found in trypsin-like proteases. The CysP1 and VpR proteases are predicted by SignalP analysis to contain intrinsic signal sequences, MGKTSKYVTAAALCSTIVMGGLHASSVSYA and MKKTTSILLSMALVFSSFGALSAHA, respectively. Deletion of these proteases by the procedure to be described below produced strain BH480 with genotype: *spo0A-*, pXO1-, pXO2-, *nprB-*, *tasA-*, *cam-*, *inhA1-*, *inhA2*, *mmpZ-*, *cysP1-*, *vpR-* (S3 Table). Confirmation of the deletions is shown in S1 Fig.

The same analysis was then applied to identify proteases in the BH480 secretome, leading to the identification of proteases NprC and S41 (Panel C in S1 Fig). NprC belongs to the M36 family of Zn-dependent metalloproteases and the C-terminal processing protease S41 belongs to serine protease family S41. The SignalP algorithm predicts that NprC contains an intrinsic signal sequence, MFNKKMVAMAMTVPLVMGTLSTVSA, whereas S41 contains a strong N-terminal transmembrane anchoring domain, MVVAFLIGAGGMFAGMSL. Deletion of these proteases produced the strain BH500 with genotype: *spo0A-*, pXO1-, pXO2-, *nprB-*, *tasA-*, *cam-*, *inhA1-*, *inhA2*, *mmpZ-*, *cysP1-*, *vpR- s41-* (S3 Table).

The mass spectrometric analyses of these strains used to identify and confirm the presence of the four new proteases are summarized in panel C of S1 Fig. Two biological replicas obtained from the BH460 and BH480 secretomes were analyzed by LC MS-MS. Peptides belonging to CysP1 and VpR were identified in the BH460 secretome as noted above, and were shown to be absent in BH480. Peptides from NprC and S41 were present in BH480, from which these proteases were deleted to produce strain BH500. No proteomic data was obtained for the BH500 strain, and instead, DNA sequencing was used to confirm deletion of these two proteases (Fig 1B), as noted below.

The scheme of deletion of the proteases described above is shown in Fig 1A. Temperature sensitive vectors containing a fragment flanking the upstream end of *Δ* (pSCL, pSCFL or pSCPL) were inserted into the genome of *B*. *anthracis* as the first single cross-over event at 37°C. Subsequent treatment with the corresponding T-SSR removed the vector, retaining the target sequence and a DNA fragment that should be inserted. Both of these elements are flanked by the upstream fragment and still contain the downstream located *Δ* fragment. Temperature sensitive vectors with the fragment flanking the downstream end of *Δ* (pSCR, pSCFR or pSCPR) were inserted into the genome of *B*. *anthracis* as the second single cross-over event at 37°C. The last treatment with the T-SSR produces a structure containing a DNA fragment that was inserted instead of deleted and a target for the T-SSR. Confirmation for the protease gene deletions is shown on Fig 1B.

### *B*. *anthracis* strain BH500 produces recombinant proteins in high yield and purity

The *B*. *anthracis* toxin components PA, LF, and EF were purified from transformants of BH500 containing plasmids pYS5, pSJ115, and pSJ136EFOS, respectively (Fig 2), in excellent yield and purity. While useful amounts of these proteins could be obtained from strain BH460 [9], use of BH500 typically gave better results. For example, LF yields after purification were often > 50 mg of pure, functional protein per liter of culture. LF made from this strain has been used successfully in many projects, such as the ongoing tumor-targeting work our laboratory has previously described [19].

Strain BH500 was also used to express HtrA. This serine protease is a member of the better-known DegP family of Gram-negative cell-surface proteases that play a key role in protein secretion, folding, and quality control. In the case of *B*. *anthracis*, HtrA has been implicated as a virulence factor [20]. The gene and its promoter were cloned into pUTE29 and transformed into BH500. Surprisingly, we found strong expression of a protein in the supernatant of the BH500 (pUTE29-*htrA*) transformant, and this was easily purified. The calculated molecular mass of HtrA is 43.9 kDa. However, analysis of the gel (Fig 2), demonstrated that the secreted HtrA migrates as if it were less than 40 kDa. The purified protein was transferred to a polyvinylidene difluoride membrane and subjected to N-terminal sequence determination. The sequence of the first 12 residues was VNKAKNETDLPG, indicating the loss of a 76-amino acids pro sequence. This sequence contains a strong transmembrane domain (S2 Fig), consistent with the anchoring of *Bacillus* HtrA proteases by their N-terminal domains [21]. It appears that overexpression increases self-processing to release the mature enzyme of 337 amino acids (35.9 kDa) that we obtained. After several chromatographic purification steps, the pure HtrA protein was obtained in a yield of 4 mg per liter of culture supernatant.

### T-SSRs as a tool for DNA insertion into the genome of *B*. *anthracis*

The T-SSRs can be used both for deletion of genes, as illustrated above, and for insertion of DNA into the genome of *B*. *anthracis (*Fig 1A). An example of using the T-SSRs to create *B*. *anthracis* strains containing variable numbers of *atxA* genes in the chromosome is shown in Fig 3. Four plasmids were constructed for *atxA* gene insertions: pSCF-LA, pSCF-2183R, pSC-A and pSCP-A (Fig 3A). The pSCF-LA plasmid was used to insert the first copy of *atxA* in place of *nprC* through Flp action as indicated in Fig 1B. In this case the 2183L PCR fragment flanking the *nprC* gene from the upstream side was used for the first single cross-over inserting the *atxA* gene. Subsequent treatment with Flp, the second cross-over with pSCF-2183R, and repeated Flp treatment produced strain BH490A containing a *FRT*-site downstream of the inserted *atxA*. The second copy of *atxA* gene was inserted with Cre-*loxP*. In this case the *atxA* gene of BH490A was used itself for the cross-over, inserting a second copy of the *atxA* gene. Subsequent treatment with Cre deleted the pSC vector and inserted a *loxP* between the two copies of *atxA*, producing strain BH490A2. The third copy of *atxA* was inserted with IntXO-*PSL*. In this case the *atxA* genes of BH490A2 were used for cross-over inserting a third copy of the *atxA* gene. Subsequent treatment with IntXO deleted the pSCP vector sequences and inserted *PSL* between two copies of *atxA*, producing strain BH490A3.

Confirmation of the sequential *atxA* gene insertions was obtained by PCR with primer pair 2183seqF/2183seqR (Fig 3B and 3C). BH490A, BH490A2 and BH40A3 produced PCR fragments with gel mobilities consistent with the expected sizes of 3,636, 6,726, and 9,837 bp. The presence of a small amount of an approximately 3.5-Kbp fragment in the BH490A2 and BH490A3 strains suggests that homologous recombination between *atxA* sequences occurs at a low frequency. The presence of three *atxA* gene copies in BH490A3 was also confirmed by PCR analysis and sequencing of the *atxA* intergenic regions with primer pair seqALAF/seqALAR (Fig 3B and 3D). The two seqALAF/seqALAR-generated PCR fragments of approximately 700 and 800 bp were found to contain the expected *PSL* and *loxP* sites, respectively (S3 Fig).

### Increased atxA copy number enhances pagA expression in a CO_2_-dependent manner

To confirm that the *atxA* inserts act as a positive transcriptional regulator along with CO_2_, we assayed the transcription of both the *atxA* and *pagA* genes via qPCR for strains containing 0, 1, 2 and 3 copies of the *atxA* gene. Plasmid pPAGK was constructed so as to have the *pagA* gene in an environment like that in pXO1 (Fig 4A). Thus, the transcription of *pagA* in pPAGK is under control of the full-length native *pag* promoter, with other promoter activities not being expected to influence its transcription. Experiments were performed both in air and carbon dioxide conditions. RNA integrity numbers for preparations from cultures grown in CO_2_ and air and used in the qPCR reactions averaged 9.43 (± 0.46) and 8.98 (± 0.83), respectively.

We found that as the copy number of *atxA* increased, the transcription of the gene also increased for strains grown in both air and CO_2_ (Fig 4B, left-hand panels; S4 Table). As expected, no *atxA* transcription was detected in the negative control Ames 35 ΔAtxA or in the strain without a chromosomal *atxA* insert, BH490 pPAGK. Increasing the *atxA* copy number had a positive effect on *pagA* transcription, but only when bacteria were grown in CO_2_, with no increase seen for cultures grown in air (Fig 4B, right-hand panels). BH490A3 pPAGK had significantly higher levels of *pagA* expression in CO_2_ than Ames 35 (p<0.05). The *pagA* expression of BH490 derivatives when grown in air was determined to be significantly higher than in Ames 35 (*p*<0.05), presumably due to the high copy number of plasmid pPAGK, and residual (leaky) promoter activity of its promoter in air.

To determine how the chromosomal inserts of *atxA* impacted PA production we conducted a Western blot analysis with PA antibodies on proteins secreted by the same strains and in the same conditions as the qPCR analysis. When grown in CO_2_, an increased *atxA* copy number led to an increase in PA production, but when grown in air, PA production appeared to remain constant with increasing copy number (Fig 4C). This result agrees with transcriptomic data indicating that AtxA is an enhancer of the positive effect of carbon dioxide on *pagA* expression.

## Discussion

This work demonstrates how sequential use of different T-SSRs can facilitate the deletion and insertion of DNA fragments into *B*. *anthracis*. These recombinases are shown to enable the creation of strains having improved behavior as hosts for recombinant protein production. Furthermore, strains having complex gene duplications can be created for study of transcriptional control, as occurs in *B*. *anthracis* in response to CO_2_.

Relying on a single T-SSR to make multiple gene knock-outs in a strain can produce problems. This became evident in our early work when *loxP* sites placed about 30 kbp apart led to deletion of the intervening region, to produce strain McrB3P-Mrr-LΔ30, which remained viable in spite of lacking many genes [5]. More recently, attempts to use Cre recombinase to further manipulate strain BH460, which contains five *loxP* sites [9], produced strains that grew poorly and generated undesirable deletions in the plasmids that were used for transformation. To resolve this problem, we brought into use two additional T-SSR systems: Flp-*FRT* and IntXO-*PSL*. The functionality of both systems was demonstrated previously [6, 8]. These enabled the inactivation of four additional proteases that were identified by proteomic analysis of the BH460 and BH480 culture supernatants. Successful application of the newly identified IntXO recombinase expanded the repertoire of available enzymes that are useful for advanced genome engineering of *B*. *anthracis* and that may find application in other microorganisms and in eukaryotes.

We also cloned and purified the HtrA protease that has been described as a major virulence determinant of *B*. *anthracis* [20]. However, the molecular mass of the secreted HtrA identified in this study (35.85 kDa) was lower than that of the HtrA secreted by strains described by Chitlaru et al. [20]. To clarify this discrepancy, we are working to produce antibodies to HtrA to facilitate analysis of the secretion, membrane anchoring, and release to the supernatant of this protease.

The development and implementation of three T-SSR systems for *B*. *anthracis* allowed us to produce sequential insertions of three copies of the *atxA* gene into the BH490 genome. The PCR product inserted was designed to include the dual promoter controlling the expression of *atxA* [22] and a 2.7-kb-long *atxA* mRNA that contains a terminator structure [23]. We found the *atxA* chromosomal inserts to be both transcriptionally and translationally active as demonstrated by the qPCR and Western blot data, proving the success of the T-SSRs in this complex genome editing procedure. The qPCR data also demonstrated the previously established cooperative relationship between AtxA and CO_2_ [12, 24]. The molecular mechanism by which AtxA and CO_2_ cooperatively regulate virulence gene expression in *B*. *anthracis* is still not known, but the strains and techniques presented here can serve as important tools to elucidate the relationship.

Furthermore, based on the protein production data presented here we propose that BH490A3 pPAGK is an appropriate host for production of recombinant proteins when grown in CO_2_, indicated by the increased level of PA production with more *atxA* inserts and the statistically significant increase in *pagA* gene expression. We also hypothesize that BH500 with three chromosomal inserts of *atxA* would be a more efficient host strain for recombinant protein production due to the tenth protease deletion.

## Supporting information

S1 Fig Identification of new proteases in the BH460 and BH480 secretomes. Top panel (A and B)—Casein proteolytic activities of HTP-chromatographic fractions for BH460 (A) and BH480 (B) supernatants. Axis X—fraction numbers, axis Y—arbitrary units of the activities, FT—flow through the column. Bottom panel (A and B)—SDS-PAGE electrophoresis of proteins in the peaks revealed during HTP-chromatography. (C) Heatmaps of proteases identified in BH460 and BH480 secretomes. Number of peptides found for proteases in BH460 and BH480 strains are shown.
(PPTX)

S2 Fig Amino acid sequence of HtrA protease. Transmembrane domain identified by TMpred software (http://www.ch.embnet.org/software/TMPRED_form.html) is indicated in red. Secreted part of the HtrA is shown in bold, amino acids identified by N-terminal degradation method are underlined. Molecular mass is 43.9 kDa for the whole molecule, and 35.85 kDa for secreted form.
(PPTX)

S3 Fig Confirmation of *loxP* and *PSL* sites location within *atxA* intergenic regions in strains with double and triple *atxA* genes. Primers seqALAF and seqALAR were used for sequencing of PCR fragments amplified with the same primers and demonstrated on Fig 3D. (A)The *PSL*-site was identified in the sequence of top PCR fragment (indicated in bold blue). (B) The *loxP*-site was identified in the sequence of the bottom PCR fragment (indicated in bold red).
(PPTX)

S1 Table PCR primers used in this study. The names and nucleotide sequences of all primers used in the current study.
(DOCX)

S2 Table qPCR primers and TaqMan probes used in this study. The names and nucleotide sequences of all qPCR primers and TaqMan probes used in the current study.
(DOCX)

S3 Table T-SSR target sites that replaced *B*. *anthracis* genes deleted. *LoxP*, *FRT* and *PSL* indicate that gene is replaced by this sequence.
(DOCX)

S4 Table qPCR raw data from ExpressionSuite software. Plasmid pYS5PAPK is the same plasmid as pPAGK.
(XLSX)

S5 Table Cycle threshold values of BH490A3 grown in CO_2_ and incubated with and without reverse transcriptase. Quantitative real time PCR raw C_t_ values from BH490A3 pPAGK RNA isolated using the procedure detailed in Materials and Methods. Samples without reverse transcriptase are marked in red and include RT- in sample name. C_t_ values of samples without reverse transcriptase are generally between 16 and 19 cycles higher than samples with reverse transcriptase indicating successful elimination of DNA during RNA isolation.
(XLSX)
