# Supplementary material for: Genome engineering in Bacillus anthracis using tyrosine site-specific recombinases
Source: PLoS One. 2017 Aug 22;12(8):e0183346. doi: 10.1371/journal.pone.0183346 (PMC5567495; doi:10.1371/journal.pone.0183346)
Supplement: S1 Fig — Top panel (A and B)—Casein proteolytic activities of HTP-chromatographic fractions for BH460 (A) and BH480 (B) supernatants. Axis X—fraction numbers, axis Y—arbitrary units of the activities, FT—flow through the column. Bottom panel (A and B)—SDS-PAGE electrophoresis of proteins in the peaks revealed during HTP-chromatography. (C) Heatmaps of proteases identified in BH460 and BH480 secretomes. Number of peptides found for proteases in BH460 and BH480 strains are shown. (PPTX) [file pone.0183346.s001.pptx]

## Slide 1
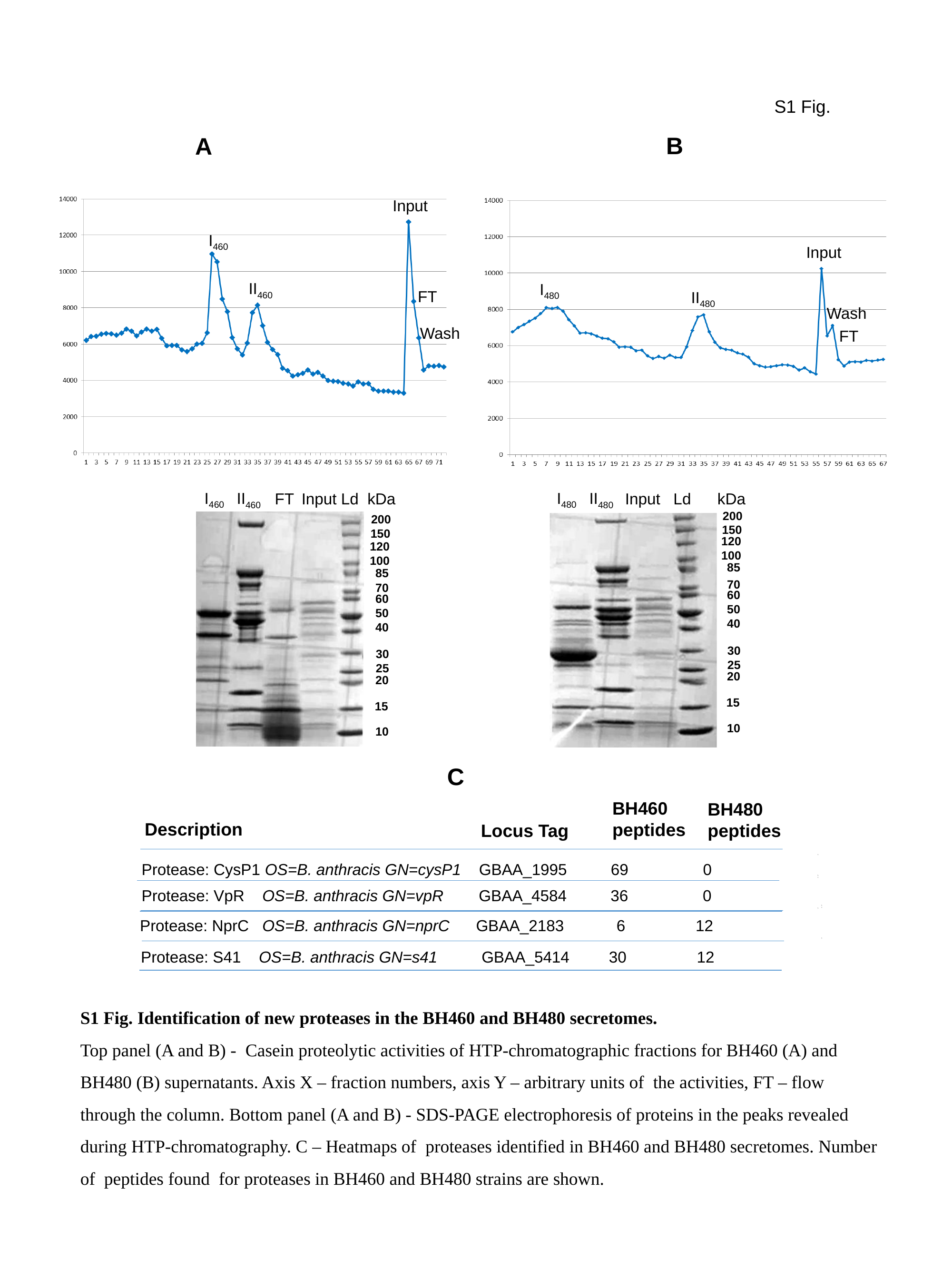

S1 Fig.
B
A
Input
I480
II480
Wash
FT
Input
I460
II460
FT
Wash
 I480
 II480
Input Ld kDa
200
150
120
100
85
70
60
50
40
30
25
20
15
10
 I460
 II460
Input Ld kDa
 FT
200
150
120
100
85
70
60
50
40
30
25
20
15
10
C
BH460
peptides
BH480
peptides
Description
Locus Tag
 Protease: CysP1 OS=B. anthracis GN=cysP1 GBAA_1995 69 0
 Protease: VpR OS=B. anthracis GN=vpR GBAA_4584 36 0
 Protease: NprC OS=B. anthracis GN=nprC GBAA_2183 6 12
 Protease: S41 OS=B. anthracis GN=s41 GBAA_5414 30 12
S1 Fig. Identification of new proteases in the BH460 and BH480 secretomes.
Top panel (A and B) - Casein proteolytic activities of HTP-chromatographic fractions for BH460 (A) and BH480 (B) supernatants. Axis X – fraction numbers, axis Y – arbitrary units of the activities, FT – flow through the column. Bottom panel (A and B) - SDS-PAGE electrophoresis of proteins in the peaks revealed during HTP-chromatography. C – Heatmaps of proteases identified in BH460 and BH480 secretomes. Number of peptides found for proteases in BH460 and BH480 strains are shown.
