# Supplementary material for: Genome engineering in Bacillus anthracis using tyrosine site-specific recombinases
Source: PLoS One. 2017 Aug 22;12(8):e0183346. doi: 10.1371/journal.pone.0183346 (PMC5567495; doi:10.1371/journal.pone.0183346)
Supplement: S2 Fig — Transmembrane domain identified by TMpred software (http://www.ch.embnet.org/software/TMPRED_form.html) is indicated in red. Secreted part of the HtrA is shown in bold, amino acids identified by N-terminal degradation method are underlined. Molecular mass is 43.9 kDa for the whole molecule, and 35.85 kDa for secreted form. (PPTX) [file pone.0183346.s002.pptx]

## Slide 1
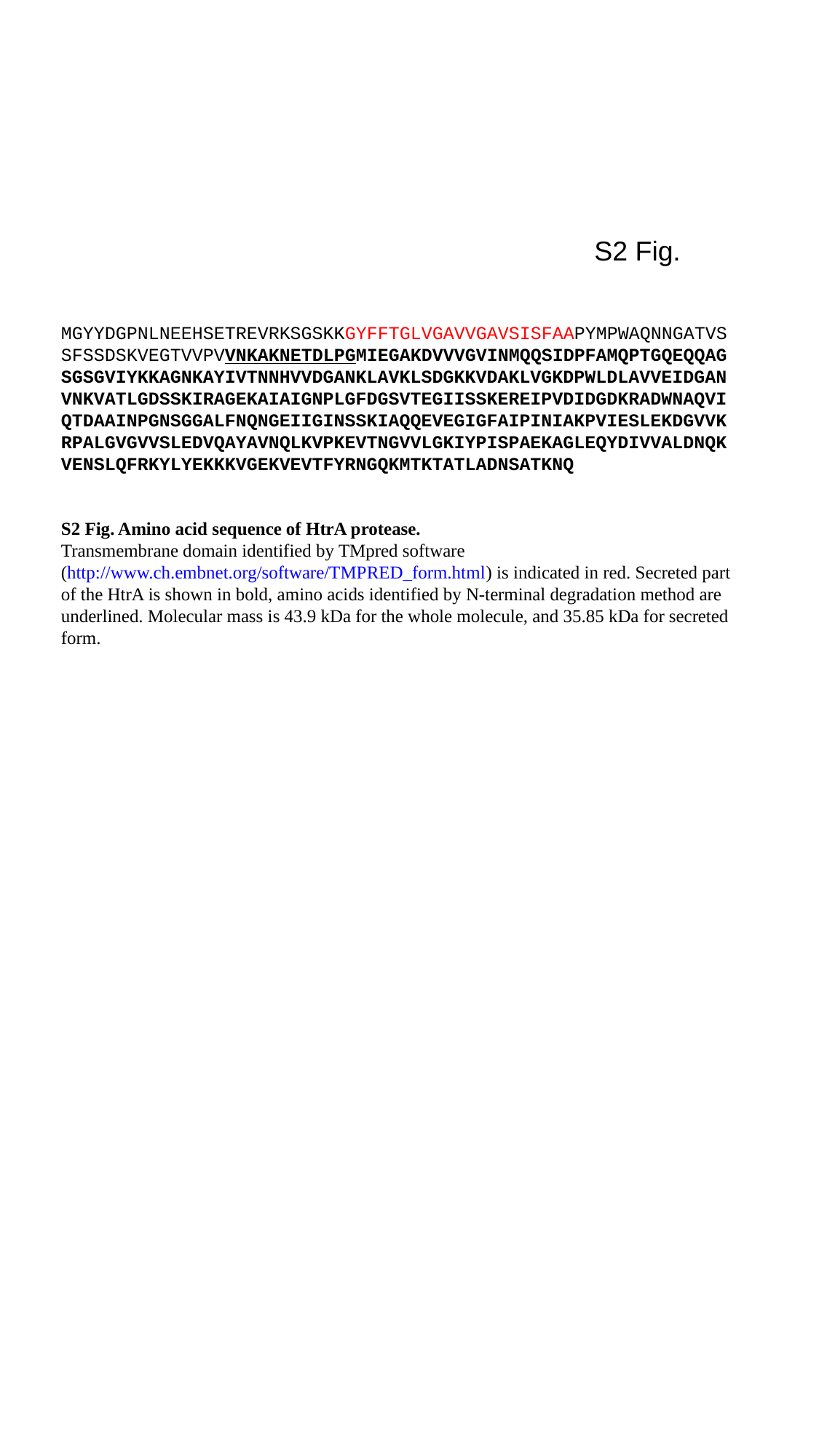

S2 Fig.
MGYYDGPNLNEEHSETREVRKSGSKKGYFFTGLVGAVVGAVSISFAAPYMPWAQNNGATVSSFSSDSKVEGTVVPVVNKAKNETDLPGMIEGAKDVVVGVINMQQSIDPFAMQPTGQEQQAGSGSGVIYKKAGNKAYIVTNNHVVDGANKLAVKLSDGKKVDAKLVGKDPWLDLAVVEIDGANVNKVATLGDSSKIRAGEKAIAIGNPLGFDGSVTEGIISSKEREIPVDIDGDKRADWNAQVIQTDAAINPGNSGGALFNQNGEIIGINSSKIAQQEVEGIGFAIPINIAKPVIESLEKDGVVKRPALGVGVVSLEDVQAYAVNQLKVPKEVTNGVVLGKIYPISPAEKAGLEQYDIVVALDNQKVENSLQFRKYLYEKKKVGEKVEVTFYRNGQKMTKTATLADNSATKNQ
S2 Fig. Amino acid sequence of HtrA protease.
Transmembrane domain identified by TMpred software (http://www.ch.embnet.org/software/TMPRED_form.html) is indicated in red. Secreted part of the HtrA is shown in bold, amino acids identified by N-terminal degradation method are underlined. Molecular mass is 43.9 kDa for the whole molecule, and 35.85 kDa for secreted form.
