# Supplementary material for: Genome engineering in Bacillus anthracis using tyrosine site-specific recombinases
Source: PLoS One. 2017 Aug 22;12(8):e0183346. doi: 10.1371/journal.pone.0183346 (PMC5567495; doi:10.1371/journal.pone.0183346)
Supplement: S3 Fig — Primers seqALAF and seqALAR were used for sequencing of PCR fragments amplified with the same primers and demonstrated on Fig 3D. (A)The PSL-site was identified in the sequence of top PCR fragment (indicated in bold blue). (B) The loxP-site was identified in the sequence of the bottom PCR fragment (indicated in bold red). (PPTX) [file pone.0183346.s003.pptx]

## Slide 1
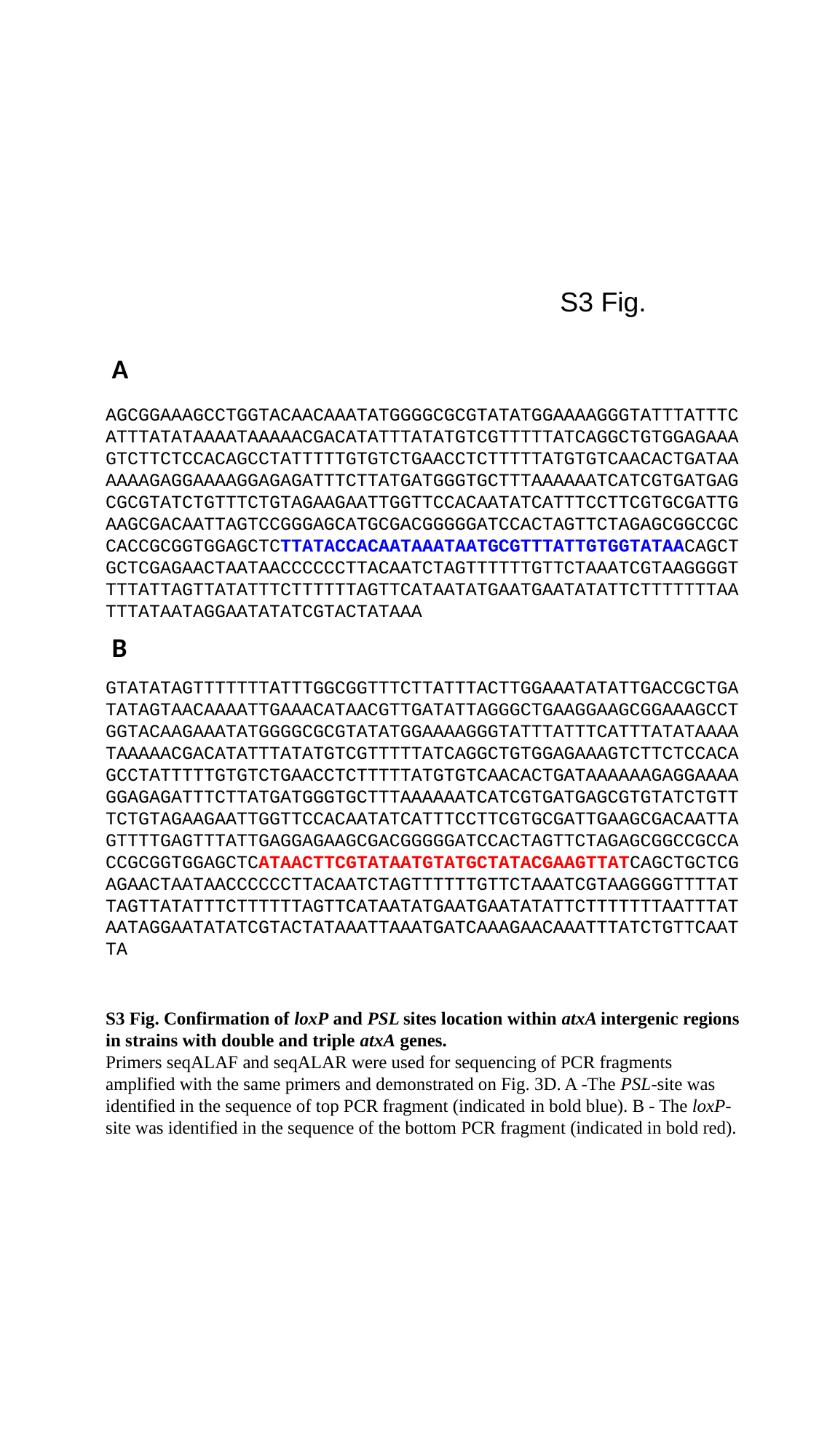

S3 Fig.
A
AGCGGAAAGCCTGGTACAACAAATATGGGGCGCGTATATGGAAAAGGGTATTTATTTCATTTATATAAAATAAAAACGACATATTTATATGTCGTTTTTATCAGGCTGTGGAGAAAGTCTTCTCCACAGCCTATTTTTGTGTCTGAACCTCTTTTTATGTGTCAACACTGATAAAAAAGAGGAAAAGGAGAGATTTCTTATGATGGGTGCTTTAAAAAATCATCGTGATGAGCGCGTATCTGTTTCTGTAGAAGAATTGGTTCCACAATATCATTTCCTTCGTGCGATTGAAGCGACAATTAGTCCGGGAGCATGCGACGGGGGATCCACTAGTTCTAGAGCGGCCGCCACCGCGGTGGAGCTCTTATACCACAATAAATAATGCGTTTATTGTGGTATAACAGCTGCTCGAGAACTAATAACCCCCCTTACAATCTAGTTTTTTGTTCTAAATCGTAAGGGGTTTTATTAGTTATATTTCTTTTTTAGTTCATAATATGAATGAATATATTCTTTTTTTAATTTATAATAGGAATATATCGTACTATAAA
B
GTATATAGTTTTTTTATTTGGCGGTTTCTTATTTACTTGGAAATATATTGACCGCTGATATAGTAACAAAATTGAAACATAACGTTGATATTAGGGCTGAAGGAAGCGGAAAGCCTGGTACAAGAAATATGGGGCGCGTATATGGAAAAGGGTATTTATTTCATTTATATAAAATAAAAACGACATATTTATATGTCGTTTTTATCAGGCTGTGGAGAAAGTCTTCTCCACAGCCTATTTTTGTGTCTGAACCTCTTTTTATGTGTCAACACTGATAAAAAAGAGGAAAAGGAGAGATTTCTTATGATGGGTGCTTTAAAAAATCATCGTGATGAGCGTGTATCTGTTTCTGTAGAAGAATTGGTTCCACAATATCATTTCCTTCGTGCGATTGAAGCGACAATTAGTTTTGAGTTTATTGAGGAGAAGCGACGGGGGATCCACTAGTTCTAGAGCGGCCGCCACCGCGGTGGAGCTCATAACTTCGTATAATGTATGCTATACGAAGTTATCAGCTGCTCGAGAACTAATAACCCCCCTTACAATCTAGTTTTTTGTTCTAAATCGTAAGGGGTTTTATTAGTTATATTTCTTTTTTAGTTCATAATATGAATGAATATATTCTTTTTTTAATTTATAATAGGAATATATCGTACTATAAATTAAATGATCAAAGAACAAATTTATCTGTTCAATTA
S3 Fig. Confirmation of loxP and PSL sites location within atxA intergenic regions in strains with double and triple atxA genes.
Primers seqALAF and seqALAR were used for sequencing of PCR fragments amplified with the same primers and demonstrated on Fig. 3D. A -The PSL-site was identified in the sequence of top PCR fragment (indicated in bold blue). B - The loxP-site was identified in the sequence of the bottom PCR fragment (indicated in bold red).
