# Supplementary material for: Genome engineering in Bacillus anthracis using tyrosine site-specific recombinases
Source: PLoS One. 2017 Aug 22;12(8):e0183346. doi: 10.1371/journal.pone.0183346 (PMC5567495; doi:10.1371/journal.pone.0183346)
Supplement: S1 Table — The names and nucleotide sequences of all primers used in the current study. (DOCX) [file pone.0183346.s004.docx]

**S1 Table. PCR primers used in this study.**

| **Primer** | **Sequence *^a^* (5'-3') (location)*** | **Relevant property** | **Site** |
| --- | --- | --- | --- |
| 1995LL | ACTGCTCGAGTGGGCTGACACATTTAAAAG | Primer pair to amplify left fragment of *cysP1* gene to clone it into pSCF | XhoI |
| 1995LR | ACTGACTAGTAGTTGAACAAAGTGCGGCAG |  | SpeI |
| 1995RL | ACTGCTCGAGAATGAAATAAACTGGCCAAAAGGTG | Primer pair to amplify right fragment of *cysP1* gene to clone it into pSCF | XhoI |
| 1995RR | ACTGACTAGTCGGGAAAAACTTCAAATCCA |  | SpeI |
| 2183LL | ACTGGTATACGCATTCTTTAAAAGTAAAACAGCCG | Primer pair to amplify left fragment of *nprC* gene to clone it into pSCF | BstZ17I |
| 2183LR | ACTGCTCGAGCCACTAGGCAATCATTCTTTACAAA |  | XhoI |
| 2183RL | ACTGCCCGGGATTTAGCGATGGAGCAAAAGCA | Primer pair to amplify right fragment of *nprC* gene to clone it into pSCF | XmaI |
| 2183RR | ACTGGAGCTCCGATTCGTGTAATCCTTCTCCA |  | SacI |
| 4584LL | ACTGCTCGAGAAGCTGTCGGTACTGCTAAA | Primer pair to amplify left fragment of *vpR* gene to clone it into pSCF | XhoI |
| 4584LR | ACTGACTAGTCGAGTGCCATACTTAAAAGTATAGA |  | SpeI |
| 4584RL | ACTGCTCGAGATCCTTGGGAGAAAAATTACGGCATT | Primer pair to amplify right fragment of *vpR* gene to clone it into pSCF | XhoI |
| 4584RR | ACTGACTAGTCGCCAAACATTCATTCATTTCTTCT |  | SpeI |
| 5414LL | ACTGCTCGAGTGTCTTTTTAACAGCGATTA | Primer pair to amplify left fragment of *s41* gene to clone it into pSCP | XhoI |
| 5414LR | ACTGTCTAGATTCCAATAATTGCAACTCTA |  | XbaI |
| 5414RL | ACTGCTCGAGCAATTACAGTCAGCATTAAA | Primer pair to amplify right fragment of *s41* gene to clone it into pSCP | XhoI |
| 5414RR | ACTGTCTAGATCATCAGAAATTTTCTCTTG |  | XbaI |
| 1995seqF | TTGCCAGAGCTTTTCATTGA | Primer pair to verify *cysP1* gene disruption |  |
| 1995seqR | CGCTAATGAATAATCTGCCA |  |  |
| 2183seqF | AACTTCCTTTTTGTGTGCTGGA | Primer pair to verify *nprC* gene disruption and *atxA* gene insertions |  |
| 2183seqR | GCGAATAAATTCACACTAGCAT |  |  |
| 4584seqF | TGAGTGAAACGGCGTAACTT | Primer pair to verify *vpR* gene disruption |  |
| 4584seqR | TATTCCTTCAAAGCCGATAT |  |  |
| 5414seqF | TGCAACAAACTGGTTTATTCGTTGGC | Primer pair to verify *s41* gene disruption |  |
| 5414seqR | AAAGCTGTTCGCAGTTCAAACCAAAT |  |  |
| 146F | ACGTCTCGAGAACTAATAACCCCCCTTACAATCTA | Primer pair to amplify *atxA* gene to clone it into pGEM-T Easy | XhoI |
| 146R | ACGTGACGTCGCTTCTCCTCAATAAACTCAAAACT |  | ZraI |
| seqALAF | TGTATTCCTTTGTAGCAAGTGAATG | Primer pair to verify second and third copies of *atxA* gene insertions |  |
| seqALAR | GAATGCAAAGTGGTAATTGAACAGA |  |  |
| pagPF | GAGAGTACGTATCTAGATGAAGATGTAAATCAAGCACTATCTGGATATATGC | Primer pair to amplify *pagA* promoter to clone it into pGEM-T Easy | SnaBI |
| pagPR | GCTAAGCTTTATTAATCACTTCTTGGTCATCTACCC |  | HindIII |
| HAF | ACTGCTGCAGGCGATGATCGAACGGTTGAT | Primer pair to amplify *htrA* gene to clone it into pGEM-T Easy | PstI |
| HAR | ACTGGGATCCCGTACGAGTTGGGTTTTCAA |  | BamHI |

*Restriction enzyme recognition sites are underlined.
