# Supplementary material for: Genome engineering in Bacillus anthracis using tyrosine site-specific recombinases
Source: PLoS One. 2017 Aug 22;12(8):e0183346. doi: 10.1371/journal.pone.0183346 (PMC5567495; doi:10.1371/journal.pone.0183346)
Supplement: S2 Table — The names and nucleotide sequences of all qPCR primers and TaqMan probes used in the current study. (DOCX) [file pone.0183346.s005.docx]

**S2 Table. qPCR primers and TaqMan probes used in this study.**

| **Gene** | **Oligonucleotide type** | **Sequence** | **Amplicon size** |
| --- | --- | --- | --- |
| *pagA* | Forward primer | TGCATGCGTCGTTCTTTGAT | 73 bp |
|  | Reverse primer | GCGACCGTACTTGAATTCGAA |  |
|  | Probe | TTGGTGGGAGTGTATCT |  |
| *atxA* | Forward primer | GAAATATCGGGAAGAGAACAAGATG | 84 bp |
|  | Reverse primer | GGGAAACGGCCAATAATCATT |  |
|  | Probe | CCGCGTATTTATGTTAAGGT |  |
| *rpoB* | Forward primer | AACCATTCGATAACCGTGTATCTG | 65 bp |
|  | Reverse primer | GTCAACCATGTGCGCAAGTT |  |
|  | Probe | TGGTGTCATGTATATGATC |  |
| *gyrB* | Forward primer | GGTGACTCTGCCGGTGGAT | 55 bp |
|  | Reverse primer | TCGCTTGGAAGTGACGATCA |  |
|  | Probe | AGCAAAGCAAGGGC |  |
| *dnaJ* | Forward primer | TGGTCAACAAATCCGTGTATCTG | 58 bp |
|  | Reverse primer | TGCTGGTCCGCCGTTTA |  |
|  | Probe | AAAAGGTGAAGCGGGC |  |
