# Supplementary material for: Genome engineering in Bacillus anthracis using tyrosine site-specific recombinases
Source: PLoS One. 2017 Aug 22;12(8):e0183346. doi: 10.1371/journal.pone.0183346 (PMC5567495; doi:10.1371/journal.pone.0183346)
Supplement: S3 Table — LoxP, FRT and PSL indicate that gene is replaced by this sequence. (DOCX) [file pone.0183346.s006.docx]

**S3 Table. T-SSR target sites that replaced *B. anthracis* genes deleted.**

| **Strain name →** | **BH460** | **BH480** | **BH490** | **BH500** |
| --- | --- | --- | --- | --- |
| Gene alterations |  |  |  |  |
| Spo0A (BA_4394) | *LoxP** | *LoxP* | *LoxP* | *LoxP* |
| NprB- (BA_0599) | *LoxP* | *LoxP* | *LoxP* | *LoxP* |
| TasA- (BA_1298) | *LoxP* | *LoxP* | *LoxP* | *LoxP* |
| Cam- (BA_1290) | *LoxP* | *LoxP* | *LoxP* | *LoxP* |
| InhA1- (BA_1295) | *LoxP* | *LoxP* | *LoxP* | *LoxP* |
| InhA2- (BA_0672) | *LoxP* | *LoxP* | *LoxP* | *LoxP* |
| MmpZ- (BA_3159) | *LoxP* | *LoxP* | *LoxP* | *LoxP* |
| CysP1 (BA_1995) |  | *FRT* | *FRT* | *FRT* |
| VpR (BA_4584) |  | *FRT* | *FRT* | *FRT* |
| NprC (BA_2183) |  |  | *FRT* | *FRT* |
| S41 (BA_5414) |  |  |  | *PSL* |
| Total number proteases inactivated | Six | Eight | Nine | Ten |

**LoxP*, *FRT* and *PSL* indicate that gene is replaced by this sequence.
